# The Poor-Law Infirmary

**Published:** 1907-06-15

**Authors:** 


### THE POOR-LAW INFIRMARY.

### SOME ESSENTIALS OF ECONOMICAL WORKING.

### Storage Accommodation.

Among the more recently built Poor-law Infirmaries, the importance of adequate storage space is being recognised. At the new Hammersmith Infirmary and Workhouse at Wormwood Scrubs the accommodation is ample, and should facilitate an economical use of all the goods, both perishable and other. The general plan of arrangement leaves, however, much to be desired still, and there is scope for the steward to exercise all his powers of organisation before his domain can be considered an unqualified success. A grave defect has been made architecturally in planning the larder to lead direct out of the scullery, in which several large boilers are kept constantly in use. Even supposing the larder door to be kept always shut, a thing never known in any kitchen yet, however ubiquitous the cook, the hot air cannot fail to be sucked under the door, and every effort to maintain a low temperature in the larder may be automatically frustrated. The best-planned store rooms in connection with Poor-law work of which we have knowledge are at Glasgow. At Stobhill, the hospital of the Glasgow Parish Council, the general store is large, airy, and well lit from the roof, a gallery running round three sides of it, where light articles are stored, while around it are a number of small stores for bread, beef, milk, oils, etc. There are also two distributing rooms or shops, where patients or the attendants come for stores. These have been arranged as far as possible to represent village shops. In this way the general store has been so planned as to afford ample space, and the utmost facilities for the receipt, storage, and distribution of every kind of article required in the hospital.

### The Larder.

In most Poor-law infirmaries this does not exist. It is the custom to requisition each day exactly what is required, and neither expect nor desire that anything shall remain over. No official in an infirmary where this practice prevails is conscious of its grave disadvantages. But when it is borne in mind that the kitchen is the source and origin of waste, and that under this system the cook, often with outside workers receiving odd meals and mutely pleading for doles, has every motive for clearing the dishes and none for storing a surplus, it is apparent that it is attended with danger to true economy. The excessive inconvenience of not having enough can hardly fail to exert an influence in keeping the daily requisition just over the border, and this is the direction in which more than anywhere else the most careful precautions are needed in dealing with large numbers. In brief, the system of requisitioning for the day's requirements and providing no proper accommodation for what remains over, needs almost superhuman vigilance and rectitude to make it a tolerable success, and cannot under the best conditions be expected to yield the best results.

### The Issuing of Stores.

The weighing out of rations has reached the standard of a fine art under the Poor-law. Everybody from the resident medical officer and the matron down to the motherless baby, is entitled to his or her irreducible minimum. The steward thinks in half ounces, and the official recipes for patients' rations with their cautious fractions only break loose with a kind of jovial irresponsibility when they record such items as water—a sufficiency; salt—a sufficiency. These carefully prescribed quantities are a survival, as we have before observed, from times when the State felt it necessary to guard the poor from the effects of local indifference. It is the opinion of some of the wisest economists in the field that one of the great bars to economy in Poor-law administration at the present day is constituted by the Local Government regulations with regard to the weighing of rations. The allowance system has long been discredited in well-managed hospitals. If it were abolished to-morrow under due precautions in Poor-law institutions, the persons responsible for their administration would find their hands set free in many directions for the exercise of sound economy and the building up of a higher standard of institution housekeeping.

### Success in the Laundry.

The supervision of the laundry is one of the many minor duties of some matrons which has a great deal to do with the comfort of every one in the institution. "Snowy linen" is a great factor in the smart appearance of the wards, and the attainment of this desideratum is well within the reach of every one who insists upon it. We can heartily recommend the latest handbook of the Power Laundry, "Two Hundred Hints for the Laundry" (Percival Marshall and Co., 1s.), to those who are dissatisfied with results. The practical information on numerous small points is admirable, but we fear that people who seek advice on "Starting a Laundry," would be disconcerted to find that the only assistance given under this heading is the sapient counsel "to take care that your machinery is so arranged as to give the greatest amount of work, with the least labour." Exactly.

THE HOSPITAL *June* 15, 1907.

